# Two-Stage Classification Model for the Prediction of Heart Disease Using IoMT and Artificial Intelligence

**DOI:** 10.3390/s22020476

**Published:** 2022-01-09

**Authors:** S. Manimurugan, Saad Almutairi, Majed Mohammed Aborokbah, C. Narmatha, Subramaniam Ganesan, Naveen Chilamkurti, Riyadh A. Alzaheb, Hani Almoamari

**Affiliations:** 1 Industrial Innovation & Robotics Center, Faculty of Computers and Information Technology, University of Tabuk, Tabuk 47512, Saudi Arabia; s.almutairi@ut.edu.sa (S.A.); m.aborokbah@ut.edu.sa (M.M.A.); narmatha@ut.edu.sa (C.N.); 2 Department of Electrical and Computer Engineering, Oakland University, Rochester, MI 48309, USA; ganesan@oakland.edu; 3 Department of Computer Science and IT, La Trobe University, Melbourne 3086, Australia; n.chilamkurti@latrobe.edu.au; 4 Faculty of Applied Medical Sciences, University of Tabuk, Tabuk 47512, Saudi Arabia; ralzaheb@ut.edu.sa; 5 Faculty of Computer and Information Systems, Islamic University of Madinah, Medina 42351, Saudi Arabia; hani.almoamari@iu.edu.sa

**Keywords:** Internet of Medical Things, cloud, heart disease prediction, hybrid linear discriminant analysis with modified ant lion optimization, hybrid Faster R-CNN with SE-ResNet-101, medical image

## Abstract

Internet of Things (IoT) technology has recently been applied in healthcare systems as an Internet of Medical Things (IoMT) to collect sensor information for the diagnosis and prognosis of heart disease. The main objective of the proposed research is to classify data and predict heart disease using medical data and medical images. The proposed model is a medical data classification and prediction model that operates in two stages. If the result from the first stage is efficient in predicting heart disease, there is no need for stage two. In the first stage, data gathered from medical sensors affixed to the patient’s body were classified; then, in stage two, echocardiogram image classification was performed for heart disease prediction. A hybrid linear discriminant analysis with the modified ant lion optimization (HLDA-MALO) technique was used for sensor data classification, while a hybrid Faster R-CNN with SE-ResNet-101 modelwass used for echocardiogram image classification. Both classification methods were carried out, and the classification findings were consolidated and validated to predict heart disease. The HLDA-MALO method obtained 96.85% accuracy in detecting normal sensor data, and 98.31% accuracy in detecting abnormal sensor data. The proposed hybrid Faster R-CNN with SE-ResNeXt-101 transfer learning model performed better in classifying echocardiogram images, with 98.06% precision, 98.95% recall, 96.32% specificity, a 99.02% F-score, and maximum accuracy of 99.15%.

## 1. Introduction

Smart healthcare provides healthcare platforms that use gadgets such as wearable appliances, the IoT, and the mobile Internet to conveniently enter health documents and connect resources, individuals, and organizations. Smart healthcare involves a wide range of operatives, including physicians, nurses, hospitals, and research organizations. It consists of a dynamic framework with numerous dimensions, such as disease detection and prevention, evaluation and assessment, decision-making, healthcare management, and medical research. Smart healthcare includes automated networks, such as the IoT, the Internet, artificial intelligence (AI), Big Data, cloud networking, and 5G, as well as advanced biotechnology. The application of developing technology in protective policies and behavioral systems can aid in the early detection of possible health concerns and allow for the scheduling of relevant measures, such as monitoring treatments and developing new evaluations. The global smart health industry was worth USD 143.6 billion in 2019, and is expected to increase at a 16.2 percent annual pace between 2021 and 2027 [1].

Telemedicine systems, on the other hand, are quite diverse and are often designed to address a particular therapeutic purpose, such as remote heart monitoring and stroke rehabilitations. This feature of telemedicine systems makes them effective in terms of minimizing expenses and healthcare infrastructure overload, but it is a disadvantage when the number of patients and diseases increases. The IoMT can address the demand for improved scalability and genericity. Indeed, it combines the dependability and safety of conventional medical equipment with the genericity, dynamicity, and scalability of typical IoT capabilities [2]. It is a smart system platform that consists primarily of electronic circuits and sensors that gather biological signals from patients. To process the signals, a processing unit is used to transmit data over a network system, which is a permanent or temporary storage unit and a visual representation platform with AI techniques is used to make decisions at physicians’ convenience [3]. In the framework of body area networks, medical sensors and actuators are employed as wearable devices in the IoMT. Instead of admitting patients to hospitals, these technologies can continuously monitor patients’ health in real time, while also providing them with greater mobility and physical flexibility [4].

**Figure 1:**
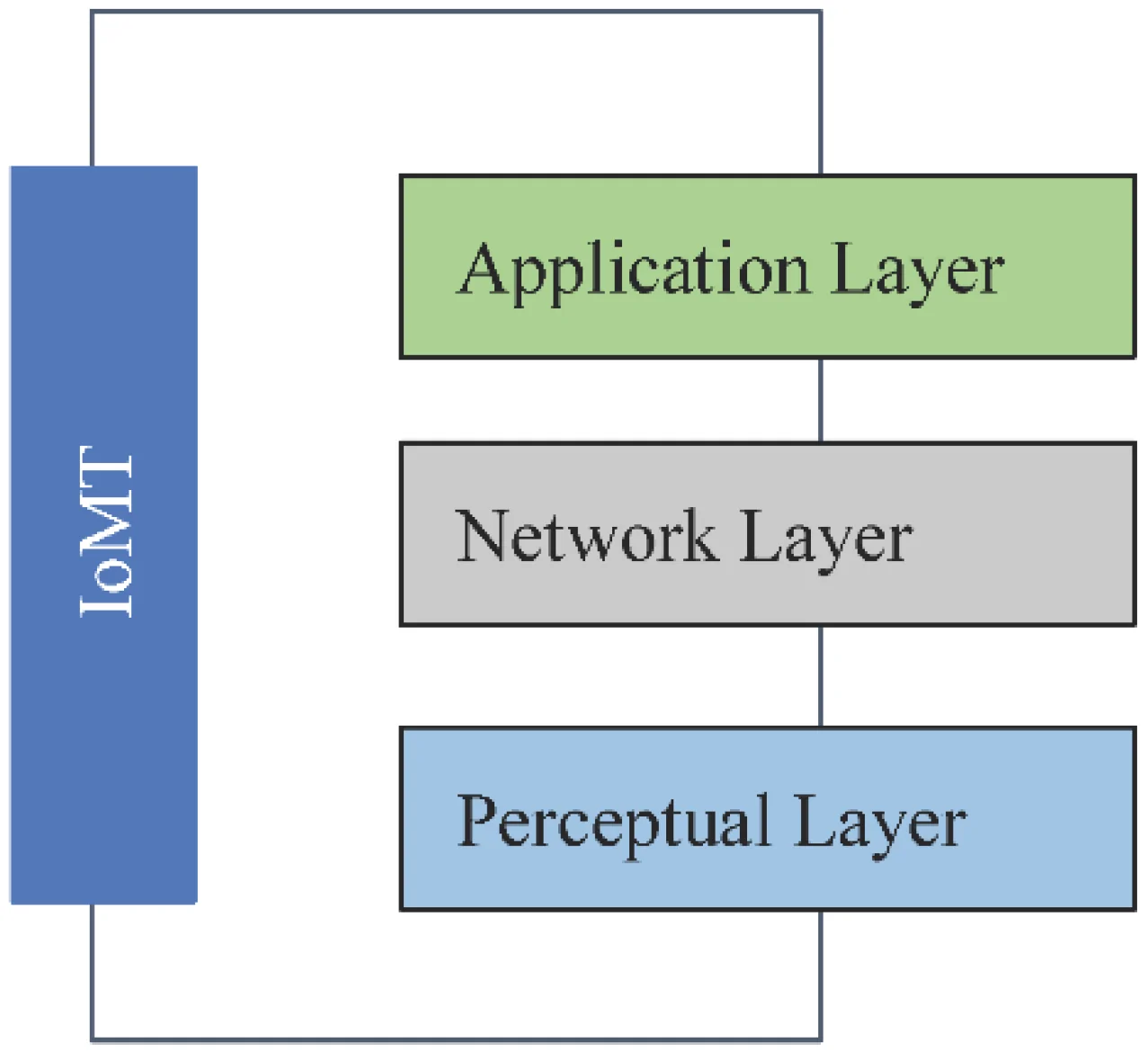
General architecture of the Internet of Medical Things.

The IoMT is the medical field’s focused manifestation of IoT technology. Figure 1 depicts the conventional three-tier design of an IoT application, called the application, network, and perception layers. The IoMT focuses on the perception layer, which is primarily separated into two sublayers: data access and data acquisition. 

In the IoMT, to accomplish the identification and perception of nodes, and to gather data about people and objects, the sublayer of data acquisition operates via various types of signal acquisition equipment and medical perception equipment. The data access sublayer connects the data acquired by the data acquisition sublayer to the network layer, using short-range transmission technologies such as Bluetooth, Wi-Fi, and ZigBee [5,6].

The network layer is also subdivided into two sublayers: the service and network transmission sublayers. The network transmission sublayer iss the IoMT’s backbone, somewhat like the brain and nerves of humans. It makes use of the mobile communications networks, the Internet, and various specialized networks for transferring data gathered through the perception layer in synchronous, precise, reliable, and barrier-free modes. The service sublayer is primarily responsible for the combination of heterogeneous networks, as well as the combination of multiple data types, data warehouses, descriptions, and other data. 

There are also two sublevels in the application layer: medical data and medical data decision-making applications. Patient data management, medical devices, material data management, and other medical data applications are examples of medical data applications. Patient data analysis, disease analysis, pharmaceutical analysis, diagnosis, therapy analysis, etc., are examples of applications of medical data decision-making [6].

The main motivation of this work is to develop a heart disease prediction model. Most of the previous research is based on either sensor-based data (medical signals) or medical images for classification and prediction. In the proposed model, instead of using sensor data and image data separately, sensor data and image data are combined as two stages of input. In the first stage, sensor data is used for prediction and classification. If the result is not satisfactory, based on the significance of the disease or the generated output, the second stage, using medical image data, will be used for accurate classification and prediction of disease. By implementing this two-stage classification model, precise predictions can be made for the benefit of both patients and doctors. 

The proposed research provides a model for medical data classification and prediction that employs artificial intelligence and machine learning techniques. Sensors (wearables) and datasets are essential components of the proposed model. As noted, the proposed model operates in two stages. The classification of sensor data generated by medical sensors placed on a patient’s body is the first stage, followed by the classification of echocardiogram images in the second stage. Both classification methods are carried out, and the classification results are validated to predict heart disease. 

A hybrid linear discriminant analysis with the modified ant lion optimization (HLDA-MALO) technique is used to classify sensor data. The hybrid Faster R-CNN with SE-ResNet-101 model is used for echocardiogram image classification.

This paper is organized as follows: Section 2 presents related works; Section 3 contains the proposed methodology; Section 4 provides the results and discussion; and Section 5 provides a conclusion and suggestions for future studies.

## 2. Related Works

A modified salp swarm optimization (MSSO) and an adaptive neuro-fuzzy inference system (ANFIS) were used in [7] to develop the IoMT system for the detection of heart diseases. Using the Levy flight method, this MSSO-ANFIS enhanced search capabilities. The common learning procedure in ANFIS is gradient-based and has a proclivity for being caught in local minima. MSSO has been used to enhance learning parameters to offer better outcomes for ANFIS. Using a deep learning modified neural network (DLMNN), a patient-monitoring method for heart patients that uses the IoT to aid in the diagnosis and treatment of heart disease, was proposed in [8]. In that work, a heart patient of the specified hospital was authenticated using the substitution cypher in conjunction with SHA-512. Next, the IoT sensor wearable gadget was attached to the patient’s body and sensor data was simultaneously relayed to the cloud. Using the PDH-AES approach, the sensor data was encoded and safely transferred to the cloud. Eventually, the encrypted data was decrypted, and the classification was completed.

A modified deep convolutional neural network (MDCNN) was used in [9] for heart disease prediction. In that work, a patient’s blood pressure and ECG were tracked using a smartwatch and an ECG device. The MDCNN was used to classify the received sensor data as normal or abnormal. For better results, the performance of this model might have been improved by applying a feature selection strategy. 

In [10], an autoencoder-based medical decision support system for cardiovascular disease diagnostics was proposed. PASCAL B-training and Physiobank-PhysioNet A-training heart sound datasets were used to construct the AEN’s diagnosing infrastructure. A learning-aided enhanced deep convolutional neural network (EDCNN) was used in [11] to help enhance prognostics of heart diseases. This model was designed with deep architecture in mind, encompassing an MLP model with regularization learning techniques. As a result, the reduction in features had an impact on the performance of classifiers, related to accuracy and processing time. 

In [12], an improved classifier, optimal deep learning, was used to classify cancer, brain imaging, and Alzheimer’s diseases. An optimal feature selection-based medical image classification was implemented, using a deep learning model that incorporated preprocessing, feature selection, and classification. The opposition-based crow search (OCS) method was employed to improve the classifier’s performance. The OCS approach chose the best attributes from pre-processed images for analysis; in that case, grey level and multi-texture features were chosen. Segmentation and feature reduction techniques might have been considered for better performance.

The convolutional neural network (CNN) was used in [13] for the detection of cardiovascular disease in a patient. In its early stage, the proposed technique was focused on temporal data modelling by using CNN for heart disease prediction. A feature extraction and selection method could be applied to improve performance. 

In ref. [14], a hybrid fuzzy-based decision tree method for early detection of cardiac disease, using a continuous and remote patient monitoring system, was proposed. If irrelevant and redundant structures are eliminated from the data, the structure chosen would aid in the improvement of model presentation for the classification of reduced data. 

A deep learning neural network heart disease prediction model using the Talos optimization technique was proposed in [15,16,17,18,19]. This model was evaluated using the medical data classification. With an ensemble of a deep learning model and feature fusion methods, a smart healthcare monitoring model was developed in [20] to predict heart disease. In that study, electronic health records and sensor data were used to predict heart disease. 

A back propagation neural network, with a maximum-relevance-minimum-redundancy feature extraction technique, was used in [21] to develop a heart disease prediction model. A numerical medical dataset was used for the evaluation of classification and prediction. A semi-supervised generative adversarial network was used in [22] to predict heart disease. In that study, echocardiogram images were used for the evaluation of heart disease.

This review of related works shows that almost all previous studies on heart disease prediction were based on either medical data classification or medical image classification. No previous work combined both medical data classification and medical image classification. Although these previous studies were carried out to predict heart disease, the concept of combining both medical data and medical image will improve the performance of classification and prediction and be useful in the prediction of several diseases.

## 3. Hybrid Classification Model for Heart Disease Prediction

The proposed model developed for medical data classification and prediction employs artificial intelligence and machine learning techniques. Sensors (wearables) and datasets are essential components of the proposed research. The proposed model operates in two stages. In the first stage classification of sensor data is generated by medical sensors affixed to a patient’s body, followed by the second stage, which is the classification of echocardiogram images. After each of these classification methods is carried out, the classification findings are aggregated and validated to forecast heart disease. This classification model is binary, and the findings are expressed as either the presence or absence of disease. 

In the study, sensor data was captured and sampled, with the ECG sensor data taken at 100 Hz. Data were sent to the system via Bluetooth and stored as binary and comma-separated value (.csv) files. The customized echocardiogram image data, gathered privately under the supervision of a doctor, was used for the image classification experiment. These files were kept in a cloud database. A hybrid linear discriminant analysis with the modified ant lion optimization (HLDA-MALO) technique was used to classify sensor data. The hybrid Faster R-CNN with SE-ResNet-101 model was used for echocardiogram image classification.

ECG, pulse oximeter, temperature, and blood pressure sensors, in the form of wearables, were used to collect medical data. These sensors recorded ECG data, heart rate, blood pressure, and body temperature by being placed on the human body. The data were captured and saved in the cloud using IoT technology. 

This research was conducted in two stages, each with its own set of challenges. The outcomes of the two stages were validated to predict heart disease. Users will be able to determine the impact of a condition by monitoring ECG, heart rate, and BP from the findings of stage 1. 

Users must have echocardiogram imaging to receive a complete diagnosis, as well as a doctor’s opinion. A doctor will evaluate the results of stage 1 and advise the patient of additional diagnostics that may be required in stage 2. Both the doctor and the user may monitor the data remotely. In the event of an emergency, a user must visit the hospital for medical support. 

**Figure 2:**
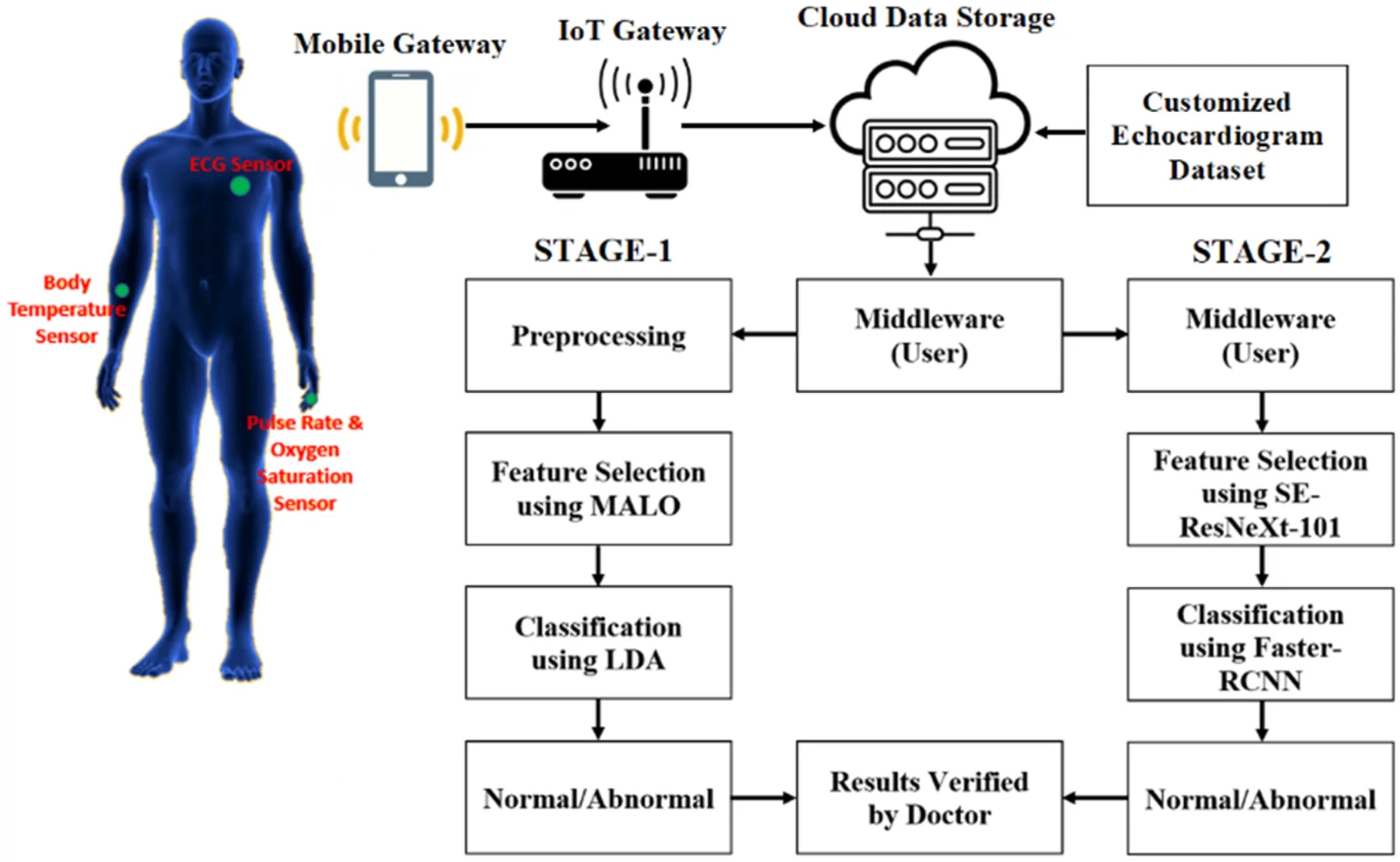
Workflow of the two-stage classification model.

Figure 2 represents the proposed model, which operates in the following manner: first, sensors (ECG, BP, and pulse oximeter) are placed on the human body to measure medical data. The sensor data obtained from patients are relevant to heart disease. The ECG sensor detects the direction of electrical impulses as they travel through the heart muscle. An abnormal heart rate is outside the range of 60 to 100 beats per minute. (Bradycardia is defined as a heart rate that is less than 60 beats per minute; tachycardia is defined as a heart rate of more than 100 beats per minute.) An arrhythmia is present if the cycle space is not even. Furthermore, if the PR interval is more than 0.2 s, the atrioventricular node is considered to be blocked.

A temperature sensor, which is an adhesive patch-based sensor with Bluetooth connectivity, was used in this research. A TMP117 high-precision digital temperature sensor combined with CC2640R2F wireless MCU reliably detects skin temperature. The average body temperature is 98.6 °F (37 °C). A temperature of more than 100.4 °F (38 °C) is considered abnormal. 

Honeywell’s 26 PC SMT pressure sensor was used in this research to assess blood pressure. Normal blood pressure is less than 120 in the systolic range and less than 80 in the diastolic range (120/80). Elevated blood pressure is defined as a systolic value greater than 120 and/or a diastolic value of less than 80. In stage 1 high BP, the systolic BP is between 130 and 139 or the diastolic BP is between 80 and 89. 

A pulse oximeter measures the SpO_2_ in the range of 70–99 percent with ±2 percent accuracy. In healthy people, the percentage saturation of oxygen assessed by a pulse oximeter ranges from 95 to 100 percent. Below 95 percent, the condition is abnormal.

The medical data collected by the sensors was transferred to the system via Bluetooth and stored as binary and comma-separated value (.csv) files. The medical data was kept in the cloud for evaluation by users and doctors. Using the stage 1 HLDA-MALO model, data stored in the cloud was accessed and processed for medical data classification. If the results of the first stage were accurate in predicting heart disease, the second stage was unnecessary. Otherwise, an echocardiogram diagnose was recommended. A heart disease dataset in the form of echocardiogram images gathered under the supervision of a doctor was also saved in the cloud for stage 2. 

For this echo image classification, a hybrid Faster R-CNN with SE-ResNet-101 model was used. Both modules were trained and evaluated using publicly available datasets from the UCI repository. During testing, the stage 1 module classified medical data and the stage 2 module separately classified medical images. The classified data and the images were finally validated by a doctor to determine whether the patient was affected by heart disease.

The first stage in the classification model is preprocessing, which is divided into three steps: replacement of missing attributes, removal of redundancies, and separation. After assessing the entirety of a patient’s age category, blood pressure, and cholesterol, the missing values of the specified attributes were added. The value was amended appropriately if the majority of a patient’s feature values matched. 

Redundancy removal minimizes data by removing irrelevant features. Patients were classified into four groups, based on the type of chest pain they were experiencing: (1) typical, (2) atypical, (3) non-anginal, and (4) asymptomatic. In stage 1, the hybrid technique (linear discriminant analysis with modified ant lion optimization) was used to classify the medical sensor data. For feature selection, the modified ant lion optimization was used, while LDA was used for classification. This research dealt with two different types of medical data, signal and image. Both types of data were analyzed independently, and the findings were validated to assess the prediction of heart disease. 

This method differs from previous efforts at predicting heart disease. The fundamental relevance of this research is that most of the previous studies focused solely on either medical image or medical signal classification. However, this research cross-validates the classification of sensor data and medical image data for the prediction of heart disease. Classification findings were verified for the purpose of predicting the presence or absence of heart disease. 

### 3.1. Hybrid Linear Discriminant Analysis with Modified Ant Lion Optimization

#### 3.1.1. Modified Ant Lion Optimization

The ant lion optimizer (ALO) is a heuristic search algorithm with no parameters, replicating the hunting approach of the ant lion in nature. Using a random walk and a “roulette wheel,” the ALO offers considerable potential for avoiding local optima stagnation. Exploration of the search space in the ALO is assured by the random selection of ant lions and the random travel of ants around them, while exploitation is guaranteed by the adaptive decreasing bounds of ant lion traps. The mathematical model of the ALO may be explained by the steps below [15]. Because ants travel in nature in a stochastic manner in search of food, the ant’s random walk may be defined in Equation (1):

(1) $$Z^{k}=[0,\;cumsum\left(2s\left(k_{1}\right)-1\right),\;cumsum\left(2s\left(k_{2}\right)-1\right),\ldots ,\;cumsum\left(2s\left(k_{n}\right)-1\right]$$

where $Z^{k}$ represents the ant’s random walk, *n* is the maximum number of iterations, *cumsum* represents the cumulative sum, *k* represents the random walk step (iteration), and *s*(*k*) is a stochastic function as defined in Equation (2):

(2) $$s\left(k\right)=\{\begin{array}{c} 1\;if\;rdm>0.5\\ 0\;if\;rdm\;\leq 0.5 \end{array}$$

where *rdm* is the random number generated using a uniform distribution within the range [0,1]. The location of each ant was normalized using a min–max normalization equation to maintain the random walk of ants inside the search space, as defined in Equation (3):

(3) $$Z_{j}^{k}=\frac{\left(Z_{j}^{k}-p_{j}\right)\left(q_{j}-r_{j}^{k}\right)}{t_{j}^{k}-p_{j}}+r_{j}^{k}$$

where $p_{j}$ is the lowest random walk of the variable *j*, $q_{j}$ is the maximum random walk of the variable *j*, $r_{j}^{k}$ is the minimum of the variable *j* at *k*th iteration, and $t_{j}^{k}$ is the maximum of the variable *j* at *k*th iteration. By these processes, an ant lion may construct a trap that is proportionate to its fitness, whereas ants must move randomly. When an ant lion detects an ant in the trap, it shoots sand outwards from the pit’s center, which cascades below the imprisoned ant that attempts to move out. To mimic the approach quantitatively, the ant’s random walk radius was reduced appropriately, as shown in Equations (4) and (5) below:

(4) $$r^{k}=\frac{r^{k}}{J}$$

(5) $$t^{k}=\frac{t^{k}}{J}$$

where $J=10^{\omega }\frac{k}{K}$ , *K* is the maximum number of iterations, and ω is a constant specified by the current iteration ( ω=2 when k>0.1K, ω=3 when k>0.5K, ω=4 when k>0.75K, ω=5 when k>0.9K, and ω=6 when k>0.95K) . Essentially, the constant ω may be used to control the exploitation accuracy. 

Levy flight (LF) distribution is a biological theory that can improve search efficiency. It was an approach to random walk with a heavy-tailed probability distribution for step lengths. The distribution of LF was used widely in evolutionary computations for tackling complicated optimization issues, due to LF’s random and dynamic features. Assume, for example, that an ant’s location is denoted by $Z_{j}$ , and the distribution of LF changes it to new condition $GZ_{j}$ . As a result, the LF distribution creates MALO, as defined in Equation (6):

(6) $$GZ_{j}=Z_{j}+\alpha ⊕Levy\left(\lambda \right)$$

where $GZ_{j}$ identifies the ant’s new state, α is the step size connected to the problem’s scales, and α is set to = 1.

To enhance the optimization capabilities of conventional ALO, the local search was added to the ALO by applying the distribution of LF for the present global best ant $Z_{l}$ , and the range over $Z_{l}$ was the most successful region for discovering optimum solutions. The fundamental approach for conducting a local search was applied initially to establish the condition, with the distribution of LF specified by $Z_{l}$ . Then, using Equation (6), the mapping value of the solution space was computed for the present iteration to improve the count of ants. Finally, each ant’s fitness value was computed, and the best ants were selected for the iterations that followed.

Feature selection is understood as a discrete optimization issue that cannot be handled directly with decimal coding. A binary coding version turns populations into the value of probability for all individuals in the binary vectors, forcing the variables to assume a value of 1 or 0. As a result, the total of ants in the ALO was based on the binary coding scheme. Each dimension in the discrete binary condition may only be represented by 0 or 1. When moving across a dimension, the relevant variables convert from 1 to 0, or vice versa. 

To implement the binary mode for the ALO, each ant’s updating mechanism was considered as being comparable to the continuous algorithm. The fundamental variation among normal and binary ALO was updating ants in the binary method, which involved switching between 0 and 1. Specifically, the coding considered the location of a new ant to be 0 or 1 with the provided probabilities, which was then updated by a condition as described in Equation (7).

(7) $$S_{j}^{k}=\{\begin{array}{c} 1\;if\;rdm<\left|\tan h\left(GZ_{j}^{k}\right)\right|\\ 0\;\;\;\;\;\;\;\;\;\;\;\;\;\;\;\;\;\;\;\;\;\;\;\;\;otherwise \end{array}$$

where tanh denotes the hyperbolic tangent functions and $S_{j}^{k}$ is the binary coding form of an ant’s location. Elitism is a significant feature of the swarm intelligence algorithm because it facilitates the best solution at any step of the optimization procedure; however, that feature was not suitable for binary coding.

Crossover requires many parent solutions and generates child solutions from entire populations. It is a comparison of the two solutions of binary coding, derived from a random move. In each iteration, the best ant lion was retained as elite. Because elite describes the fittest and best ant lion, that factor must have influenced the movement of each ant in an iteration. As a result, it appeared that ants moved around the elite and other ant lions concurrently. This is shown by way of a “roulette wheel” in Equation (8):

(8) $$Ant_{j}^{k}=Crossover\left(S_{P}^{k},\;S_{C}^{k}\right)$$

where $S_{P}^{k}$ is the random walk around the ant lion picked by the roulette wheel at iteration *k*, and $S_{C}^{k}$ is the random walk around the elite at iteration *k*. In this paper, the MALO was presented as a solution to the problem of selecting features and obtaining the ideal combinations for medical data classifications by integrating Levy flight distribution, crossover, and binary coding operations. Using Equation (6), the present global best ants were randomly dispersed in the local space, every ant was binary-coded and its location was updated, and elitism with crossover operations occurred as per Equations (7) and (8). To increase the original algorithm’s local optimization capabilities, the LF approach was merged with the ALO to form a modified ALO.

#### 3.1.2. Linear Discriminant Analysis

Linear discriminant analysis (LDA) is commonly utilized to classify patterns into two categories, although it may be expanded to identify many patterns. LDA assumes that all classes are separable linearly, and to separate the classes, a multiple linear discrimination function representing numerous hyperplanes in the feature space is created. If there are two classes, LDA draws one hyperplane and projects the data onto it in such a way that the separation of the two classes was maximized. This hyperplane is generated by taking two factors into account simultaneously, as in [16]:

- maximizing the difference between the two classes’ means; and
- minimizing diversity within each category.

LDA is a prominent pattern identification approach. There are various medical applications for LDA classifiers, such as electrocardiogram (ECG) signal analysis, lung cancer classification, and breast cancer classification. LDA seeks the optimal sets of discriminant projection vector *Y* to map the actual data space onto a low dimensional features space, by increasing the fisher criterion *I*(*Y*) that indicates that the overlap among the classes in the low dimensional features space was minimal. The $Y,\;Y^{\prime }$ and class distributions are projected in two-dimensional space. While the projected space $Y^{\prime }$ has significant class overlap (classification error), the equivalent projection *Y* has much better class separation. For example, let $Z=\left\{z_{1},\;z_{2},\ldots ,z_{N}\right\}$ represent the data collection of an N-dimensional vector. Every data point is assigned to one of the *R* object types $\left\{Z_{1},\;Z_{2},\ldots ,Z_{j},\ldots ,Z_{r}\right\}$ . The scatter matrices (i.e., the between-class scatter matrix and the within-class scatter matrix) are defined in Equations (9)–(12):

(9) $$M_{q}=\overset{R}{\underset{j=1}{\sum }}O_{j}\left(o_{j}-o\right){\left(o_{j}-o\right)}^{K}$$

(10) $$M_{y}=\overset{R}{\underset{j=1}{\sum }}O_{j}$$

where

(11) $$O_{j}=\underset{Z\in z_{j}}{\sum }\left(z-\mu_{j}\right){\left(z-\mu_{j}\right)}^{K}$$

(12) $$M_{q}=\overset{R}{\underset{j=1}{\sum }}O_{j}\left(o_{j}-o\right){\left(o_{j}-o\right)}^{K}$$

The mean of the samples in class *j* is denoted by $o_{j}$ , while the mean of all samples is denoted by *o*. LDA as a function of *Y* can be expressed as in Equation (13):

(13) $$I\left(Y\right)=\frac{\left|Y^{K}M_{Q}Y\right|}{\left|Y^{K}M_{Y}Y\right|}$$

*Y* was chosen in such a way that *I*(*Y*) was maximized. The solution $\left\{y_{j}|j=1,2,\ldots ,N\right\}$ was the collection of generalized eigenvectors related to the standard eigenvalues $\lambda_{1}\geq \lambda_{2}\geq \ldots \geq \lambda_{N}\geq 0$ in the generalized eigenvalue problem shown in Equation (14):

(14) $$M_{Q}y_{j}=\lambda_{j}M_{y}y_{j}$$

The eigenvector columns of *Y* that correspond to the highest eigenvalue $\lambda_{j}$ are represented by $y_{j}$ in this relationship. These eigenvectors are the columns of the transformation matrix *Y* for the eigenvectors $y_{j}$ , and the dimensional reduction of the data point was decreased using the transformation. The sensor data from the ECG sensors was sampled at 100 Hz. Data were sent to the system via Bluetooth and stored as binary and comma-separated value (.csv) files. 

IoMT devices and sensors are part of the IoT system. They are designed to gather medical data from remote areas. These data are collected as patient information, using IoT sensors connected to the human body.

### 3.2. Hybrid Faster R-CNN with SE-ResNeXt-101

**Figure 3:**
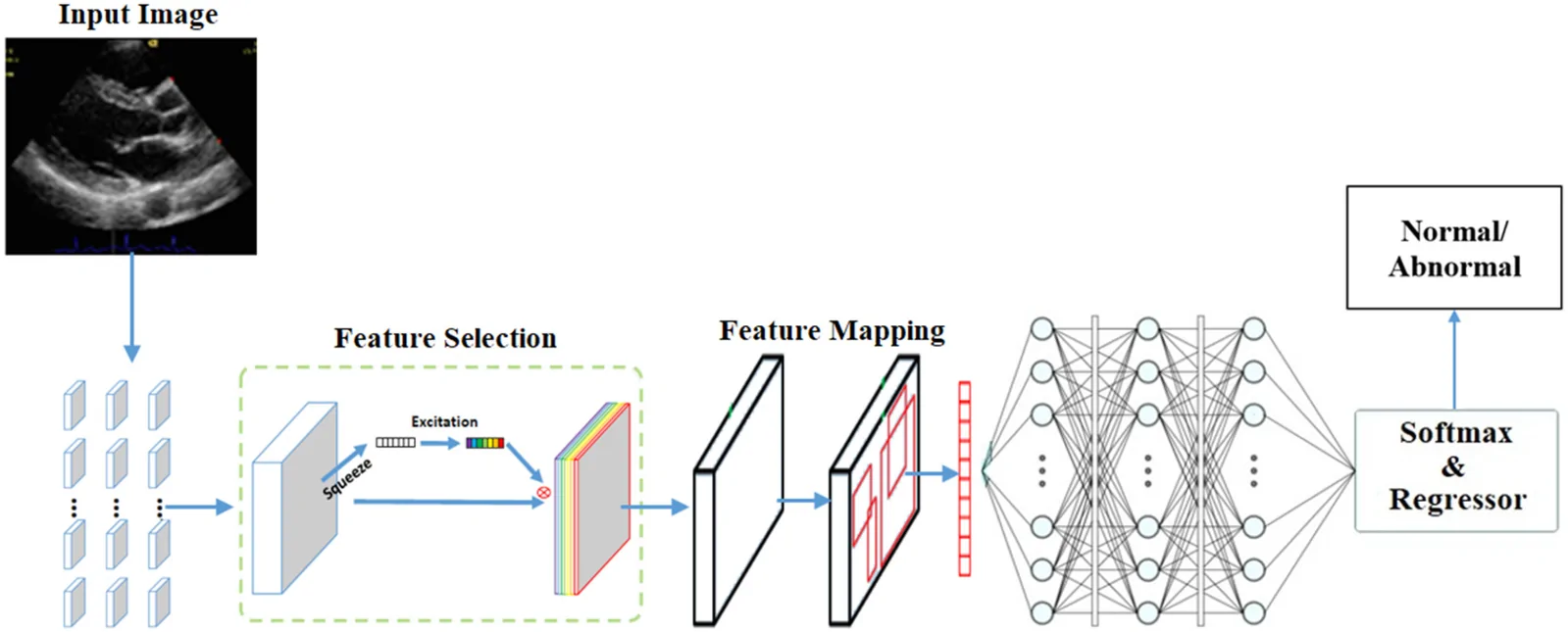
Architecture of proposed hybrid Faster R-CNN with SE-ResNeXt-101.

In stage 2, a Faster-RCNN with pretrained SE-ResNeXt-101 was implemented for echo image classification. This SE-ResNeXt-101 model was built on deep transfer learning and was designed to diagnose heart disease from classifying echocardiogram images. A pre-trained SE-ResNeXt-101 model was utilized to extract features from the input image, and the Faster-RCNN model was used for classification. The input image resolution was 224 × 224 × 3. Figure 3 represents the proposed image classification model. The SE-ResNeXt-101-32x4d was a ResNeXt101-32x4d variant with an additional squeeze-and-excite module. A squeeze-and-excitation block was the computational unit that could be formed from the transformation $E_{ks}$ that translates the input $Z\in S^{B^{\prime }\times Y^{\prime }\times R^{\prime }}$ to feature mapping $V\in S^{B\times Y\times R}$ . In the characters that follow, $E_{ks}$ was a convolutional operator and $U=\left[u_{1},\;u_{2},\ldots ,u_{R}\right]$ was the learned sets of filter kernels, where $u_{R}$ referred to the *R*th filter’s parameter. Thus, the output may be expressed as $V=\left[v_{1},\;v_{2},\ldots ,v_{R}\right]$ in Equation (15):

(15) $$v_{r}=u_{r}*Z=\overset{R^{\prime }}{\underset{m=1}{\sum }}u_{r}^{m}*Z^{m}$$

where * represents convolution, $u_{r}=\left[u_{r}^{1},u_{r}^{2},\ldots ,u_{r}^{R^{\prime }}\right]$ , $Z=\left[z^{1},z^{2},\ldots ,z^{R^{\prime }}\right],$ and $v_{r}\in S^{B\times Y}$ . $u_{r}^{m}$ is the 2D spatial kernel, representing a single $u_{r}$ channel that performs on the associated *Z* channels.

Bias terms were deleted to simplify the notation. Because the output is a total of each channel, channel dependencies were implicitly encoded in $u_{r}$ , but they were entangled with the local spatial correlations collected by the filters. Squeezing global spatial data into the channel descriptors could be used to address the issue of exploiting channel dependencies. This was accomplished by employing global average pooling to create channel-specific information. Formally, the statistic $a\in S^{R}$ was produced by decreasing *V* across its spatial dimensions B×Y , so that the *r*th element of *a* was determined in Equation (16):

(16) $$a_{r}=E_{md}\left(v_{r}\right)=\frac{1}{B\times Y}\overset{B}{\underset{j=1}{\sum }}\overset{Y}{\underset{i=1}{\sum }}v_{r}\left(j,i\right)$$

To use the data gathered during the squeeze operations, a second operation was performed with the goal of completely capturing channel-wise dependencies.

The ResNeXt module was a ResNet variation that was very similar to the inception model. Both adhere to the split-transform-merge model, except that in this variation the output of separate routes was merged by combining them, whereas in the inception model they were depth-concatenated. Experiments revealed that increasing cardinality yielded more accuracy than going deeper or broader. The split-transform-merge model was often performed by a point-wise grouped convolutional layer, which divided its input into groups of feature maps and performed normal convolution; their outputs were depth-concatenated and then fed to a 1 × 1 convolutional layer.

(17) $$e\left(z\right)=\overset{R}{\underset{j=1}{\sum }}K_{j}\left(z\right)$$

In Equation (17), *K_j_*(*z*) was a function of any type. *K_j_* projects *z* into a (optionally low dimensional), embedding and then altering it, analogous to a simple neuron. In Equation (17), *R* denotes the size of the collection of transformations to be aggregated. Cardinality is the term used to describe *R*. The number of more complex transformations is determined by the dimension of cardinality. The residual function is the aggregated transformation in Equation (18):

(18) $$f=z+\overset{R}{\underset{j=1}{\sum }}K_{j}\left(z\right)$$

where *f* was the output.

Faster R-CNN is a region proposal network (RPN) designed for object identification using region proposal methods to detect object positions. It employs the single network for the RPN operation, which produces region proposal operations, and Fast R-CNN for region classification. Faster R-CNN share whole-image convolutional features with Fast R-CNN. The RPN was a fully convolutional network that predicts object limits and performs simultaneously, making Faster R-CNN a total CNN-based model with no handmade features. Fast R-CNN uses the higher-quality region proposals provided by RPN after they have been trained end-to-end to find regions. The RPN accepted any size of input images and output images in a series of rectangular object proposals, each with an object score. The RPN first initialized n × n reference boxes (each sliding window) with distinct sizes and aspect ratios at each conv feature map point. Every sliding window was assigned a lower-dimensional vector, which was subsequently fed into two fully connected layers (box classifications and box regression layers). ReLUs were applied to the output of the n × n conv layers. Faster RCNN could test, at all stages, the very deep SE-ResNeXt-101 model on the GPU while obtaining advanced object detection accuracy on the proposed echocardiography dataset images [17,18].

The heart attack and echocardiogram datasets are collected from the UCI database. These databases serve as a historical repository of hospital-based health knowledge. All of these data sets were preserved on the cloud. The required data was processed on the cloud for ease of access. Using an ML-based method, the proposed classification model was developed to classify heart disease using two different types of medical data (signal and image). Both types of data were analyzed independently, and the findings were used to predict heart disease. 

This work differs from previous efforts in predicting heart disease. This IoT-based strategy was implemented in three phases. In the first phase, an IoT device gathered data from a patient’s body, data from the data collection, and data from the patient’s record. In the second phase, the complete cumulative knowledge was processed in the cloud. Data classification completed the disease classification in the third and final phase. The method next entered the testing phase, which entailed the use of the dataset to train the classifier for disease diagnosis. As a result, the trained classifier was prepared to evaluate the input patient’s data for accurate disease detection, and the results of the test could be made available to the user and the doctor.

## 4. Results and Discussion

The proposed work was implemented on Amazon cloud. The proposed model was tested using the MATLAB Simulink tool, version 2019a. The experiments were carried out using a PC with an Intel Core i7-10700 CPU running at 2.9 and 4.8 GHz, with 8 GB of RAM and a 64-bit Windows 10-OS. The classification and prediction of heart disease data was the primary objective of this work and was critical in this research. The proposed classifiers classified the data as indicating whether heart disease was present.

### 4.1. Dataset Description

The Cleveland dataset from the UCI repository, which is available on the Internet at http://archive.ics.uci.edu/ml/datasets.php (accessed on 21 July 2021), was used to evaluate the proposed model. Each data collection has its own instances and attributes; for example, the Cleveland dataset includes 76 characteristics and 303 records. However, only 14 characteristics from the Cleveland dataset were used for training and testing, as shown in Table 1. The entire dataset was used for training the proposed model, and the sensor data collected was used for testing the proposed model. Based on the features of the training dataset, all the information was collected using the sensor, which was used for testing. The features used in the training data were also evaluated in the testing data. 

**Figure 4:**
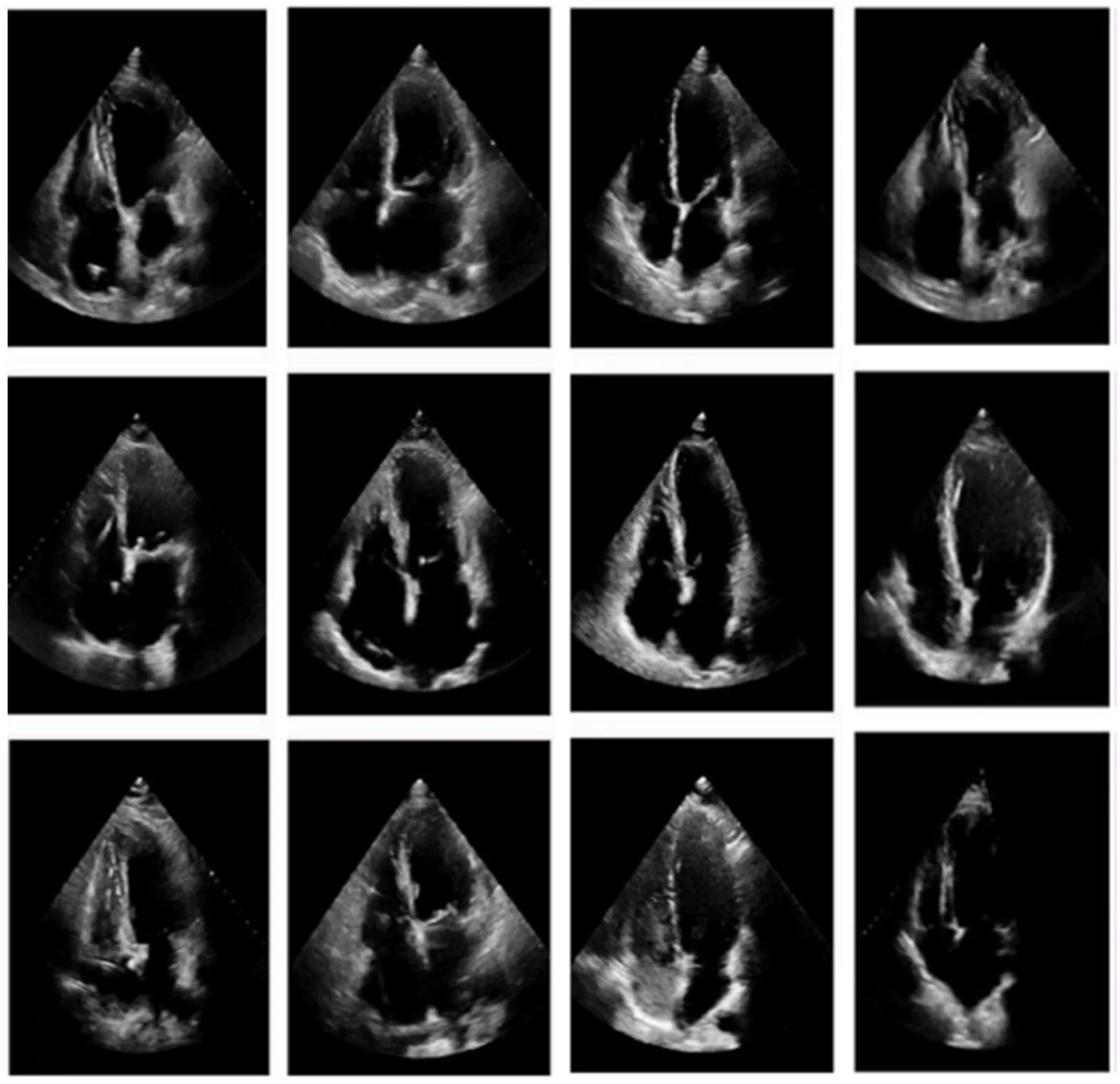
Sample images from dataset.

The description of the echocardiogram image dataset is presented in Table 2 with appropriate attributes. For the first-stage experiment, the selected features of the UCI Heart Disease dataset were used to develop and evaluate the HLDA-MALO classification model for distinguishing normal and abnormal cases. The medical sensor data collected through the proposed IoMT system was subsequently evaluated using the features considered during model development. The sensor data included measurements obtained from ECG, pulse oximeter, temperature, and blood-pressure sensors. For the second stage, the EchoNet-Dynamic dataset was used for echocardiography image classification. This dataset is publicly available and was used for the classification of echocardiographic images using the proposed hybrid Faster R-CNN with SE-ResNeXt-101 model. The echocardiography data were used to evaluate the capability of the proposed deep-learning model for distinguishing normal and abnormal cardiac images. Thus, the two stages of the proposed framework use two distinct publicly available datasets. The UCI Heart Disease dataset provides the medical attributes for the first-stage classification, whereas the EchoNet-Dynamic dataset provides the echocardiographic data for the second-stage image classification (Figure 4). The two datasets were analyzed independently according to their respective data modalities, and the resulting classification outcomes were used in the proposed two-stage heart disease prediction framework.

### 4.2. Performance Metrics

Accuracy, precision, recall, and the F-score are the output metrics shown in Equations (19)–(23). The following table compares the expected and real results, depending on these metrics:

(19) $$\mathrm{Accuracy}=\frac{TRP+TRN}{TRP+FLP+TRN+FLN}\;\%$$

(20) $$\mathrm{Precision}=\frac{TRP}{TRP+FLP}$$

(21) $$\mathrm{Recall}=\frac{TRP}{TRP+FLN}$$

(22) $$Specificity=\frac{TRN}{TRN+FLP}$$

(23) $$F-\mathrm{score}=\frac{2TRP}{2TRP+FLP+TRN}$$

*TRP:* the true positive value, which was the total correct classification in normal classes.
*FLP:* the false positive value, which was the total incorrect classification in normal classes.
*TRN:* the true negative value, which was the total correct classification in abnormal classes.
*FLN:* the false negative value, which was the total incorrect classification in abnormal classes.

To assess the performance analyses of the dataset’s normal and abnormal type cases, the HLDA-MALO algorithm was applied. Table 3 displays the accuracy, precision, recall, specificity, and F-score values for the various normal and abnormal classes in the medical data classification. In this stage 1 experiment, two distinct types of data were evaluated for classification as normal (healthy) or abnormal (unhealthy).

**Figure 5:**
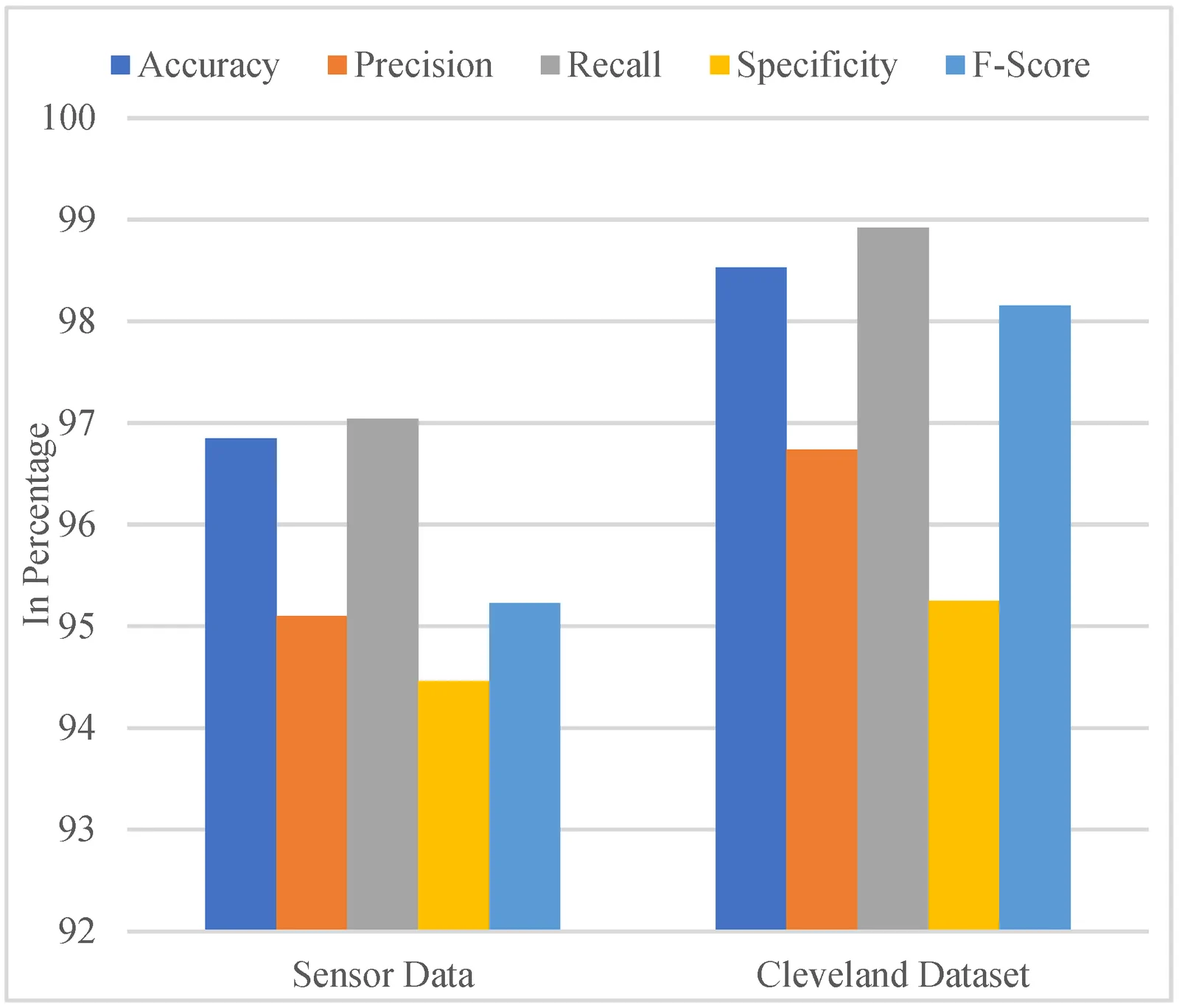
Classification of normal data.

**Figure 6:**
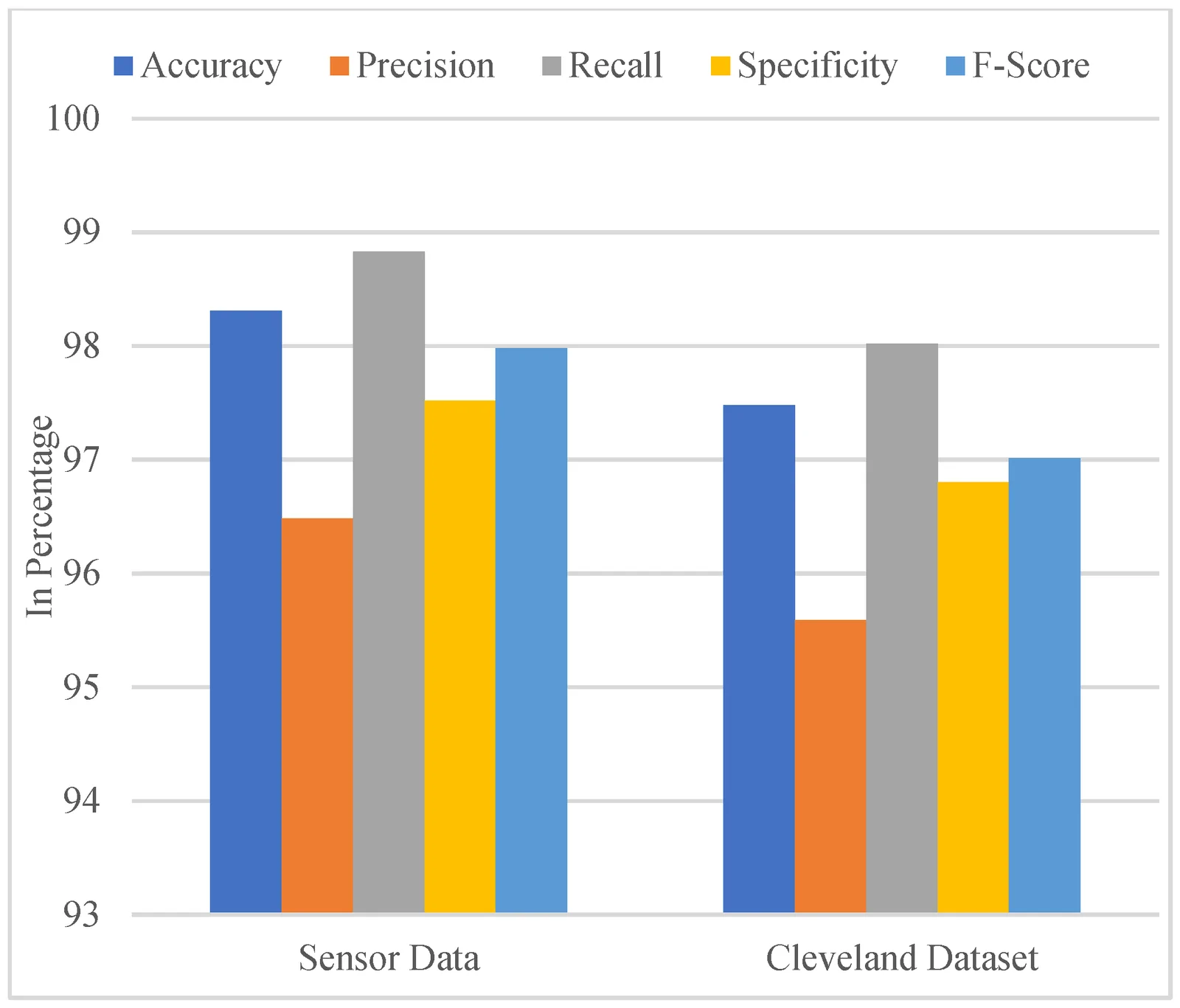
Classification of abnormal data.

The proposed model was tested in terms of accuracy, precision, recall, specificity, and F-score based on this data. Using medical sensor data gathered by sensors, the proposed HLDA-MALO technique obtained 96.85 percent accuracy, 95.10 percent precision, 97.04 percent recall, 94.46 percent specificity, and a 95.23 percent F-score in normal class classification. The HLDA-MALO approach obtained 98.53 percent accuracy, 96.74 percent precision, 98.92 percent recall, 95.25 percent specificity, and a 98.15 percent F-score on the Cleveland dataset’s normal cases. Figure 5 and Figure 6 represent the graphical plot for the performance of medical data classification. Using medical signal data gathered by sensors, the proposed HLDA-MALO technique obtained 98.31 percent accuracy, 96.48 percent precision, 98.83 percent recall, 97.52 percent specificity, and 97.98 percent F-score in abnormal class classification. The HLDA-MALO approach obtained 97.48 percent accuracy, 95.59 percent precision, 98.02 percent recall, 96.80 percent specificity, and a 97.01 percent F-score on the Cleveland dataset’s abnormal cases.

The performance study and comparison of the medical image classification model hybrid Faster R-CNN-SE-ResNeXt-101 with other known transfer learning models is shown in Table 4. The data revealed that the proposed hybrid Faster R-CNN with SE-ResNeXt-101 transfer learning model outperforms other models in all parameters, with 98.06 percent precision, 98.95 percent recall, 96.32 percent specificity, a 99.02 percent F-score, and 99.15 percent maximum accuracy.

**Figure 7:**
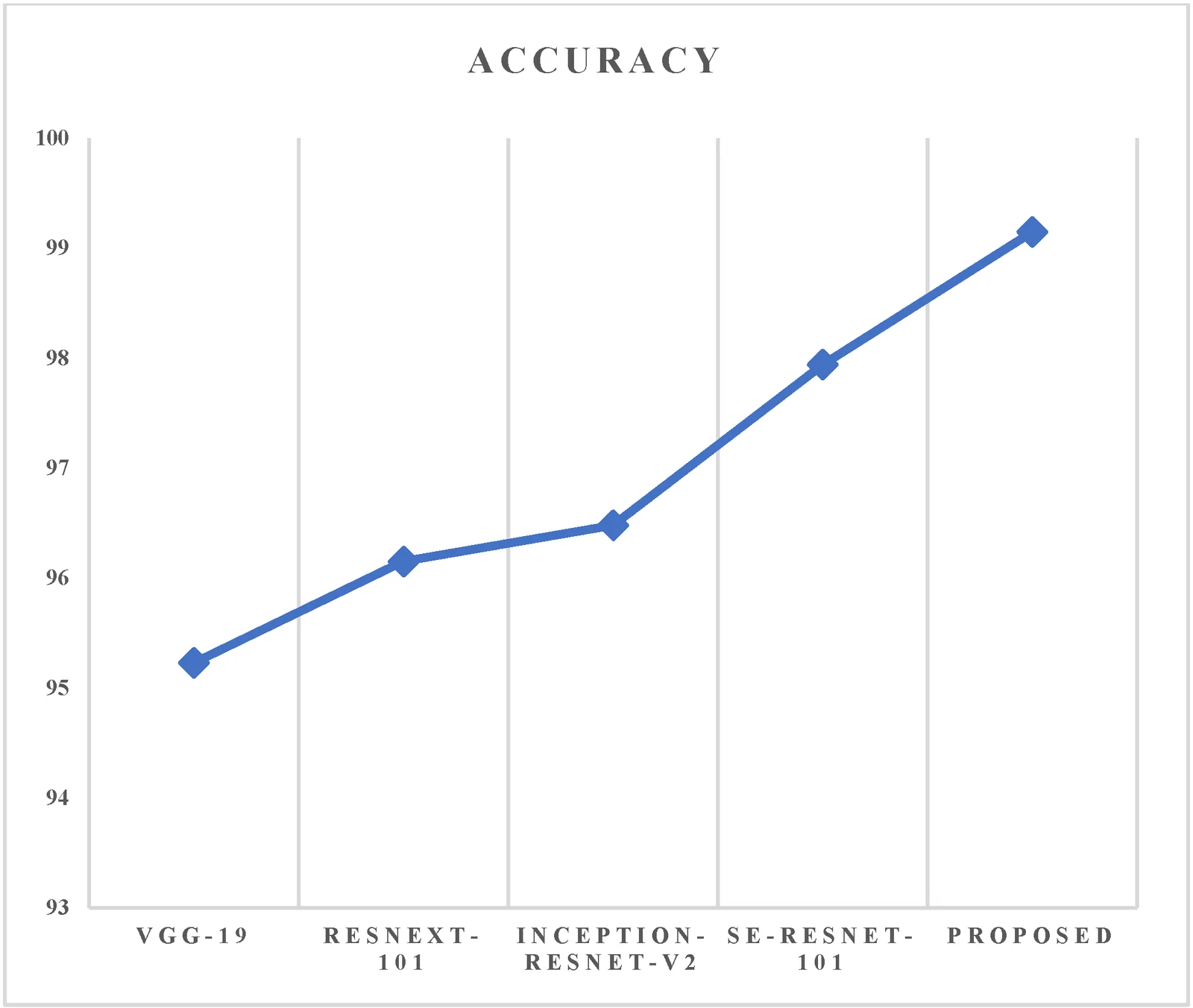
Comparison of image classification accuracy.

**Figure 8:**
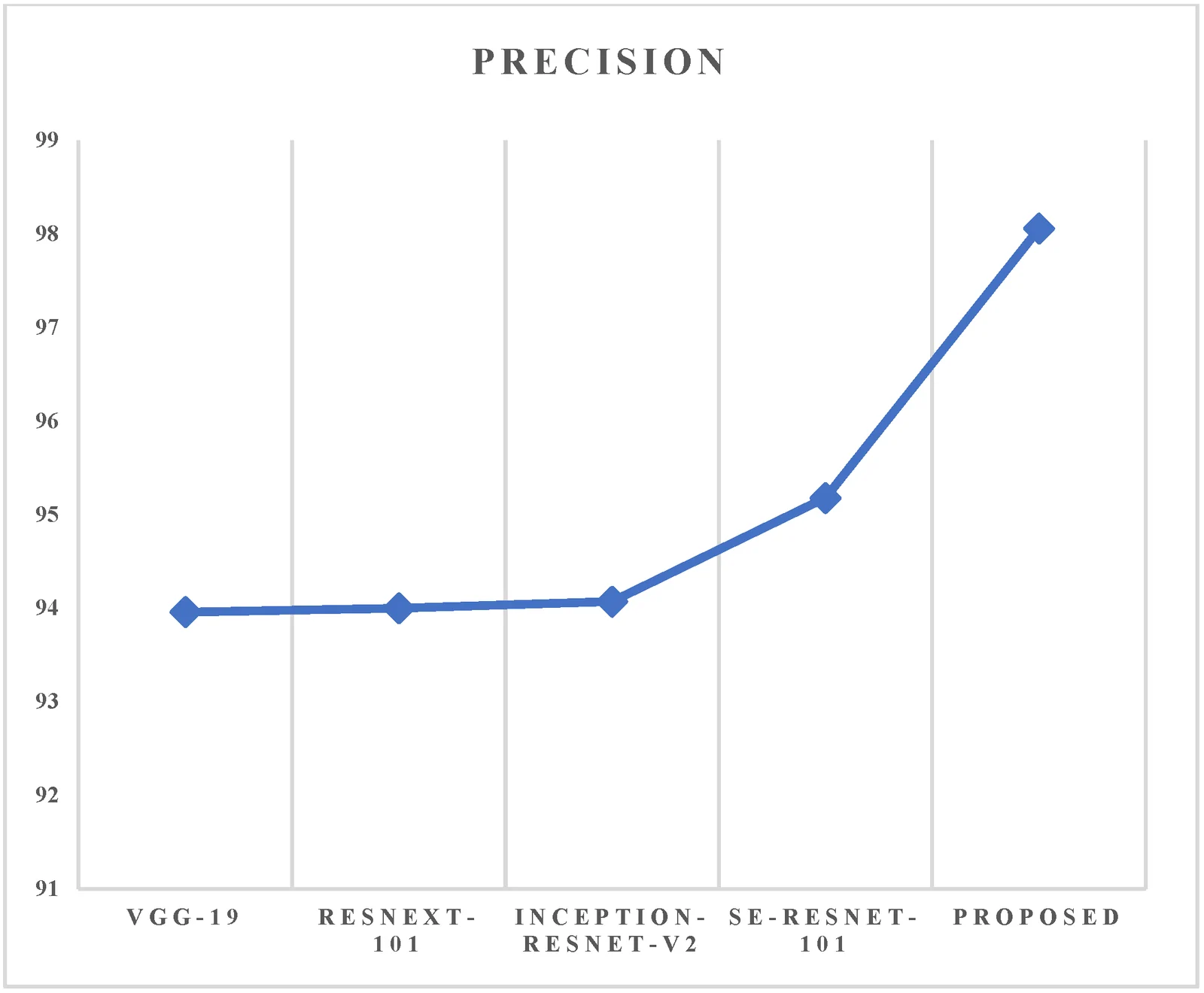
Comparison of image classification precision.

The proposed Faster R-CNN-SE-ResNeXt-101 model showed higher classification accuracy for classifying echocardiography images for heart disease prediction, as shown in Figure 7. The model achieved 99.15 percent accuracy, which is 1.2 percent to 3.9 percent higher than other approaches, such as VGG-19, ResNeXt-101, Inception-ResNet-v2, and SE-ResNet-101. The precision estimation was tabulated, indicating that the proposed model attained a higher precision value of 98.06 percent. The precision performance of the proposed model increased by 2.8 and 4.1 percent when compared to the other models, as shown in Figure 8. The reported results were computed using five-fold cross-validation, where the dataset was divided into five equal subsets. In each iteration, four folds were used for training, and one fold was used for testing, and the process was repeated five times. For example, fold accuracies such as 98.9%, 99.3%, 99.0%, 99.4%, and 99.15% were averaged to obtain the final accuracy of 99.15%. Similarly, precision, recall, specificity, and F-score values were also calculated for each fold and averaged to produce the final reported metrics.

**Figure 9:**
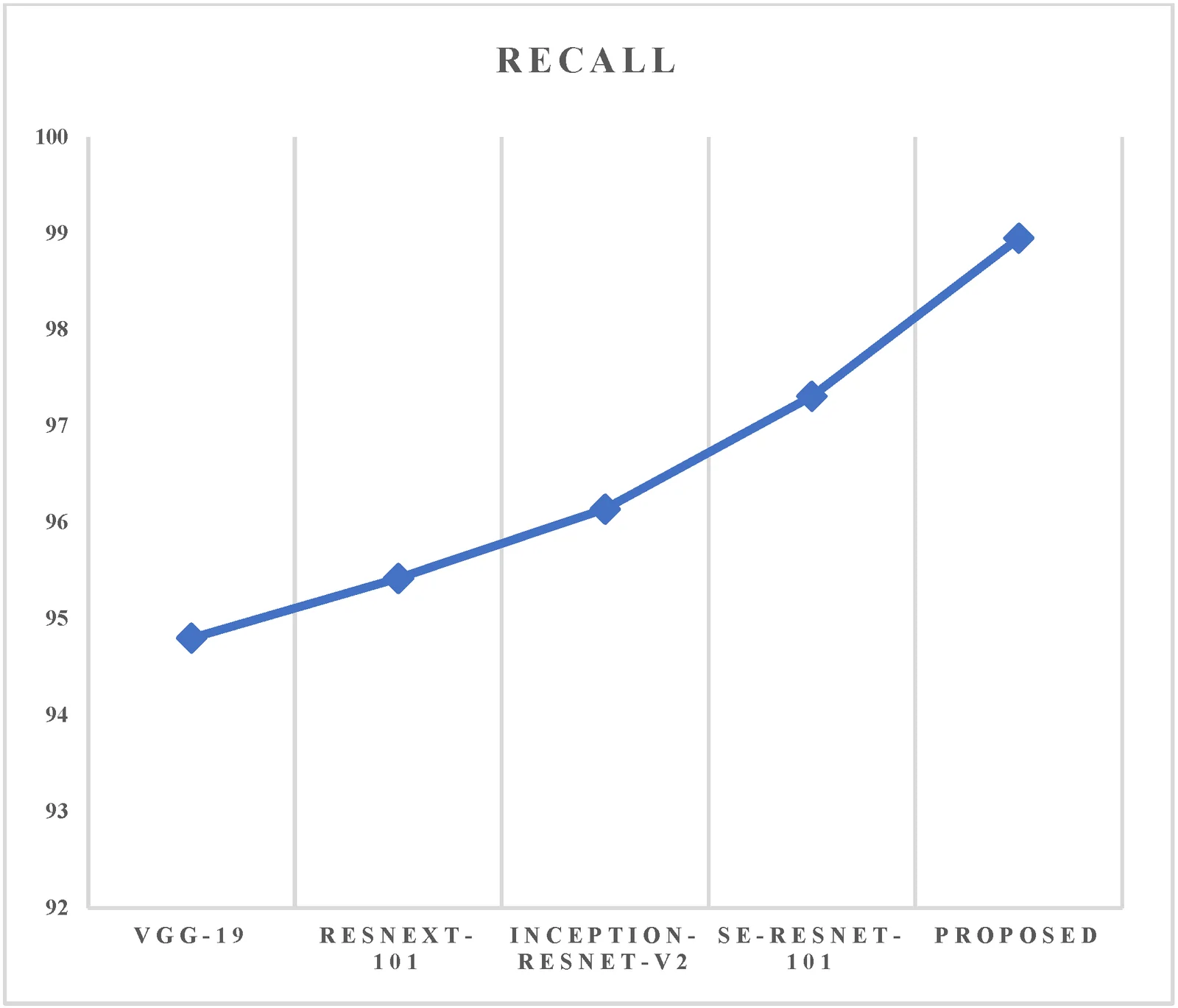
Comparison of image classification recall.

The proposed model achieved a recall or sensitivity rate of 98.95 percent as shown in Figure 9, which is 1.6 percent to 4.1 percent higher than the comparison models. In terms of performance evaluation, the proposed model outperformed the other deep learning comparison models.

**Figure 10:**
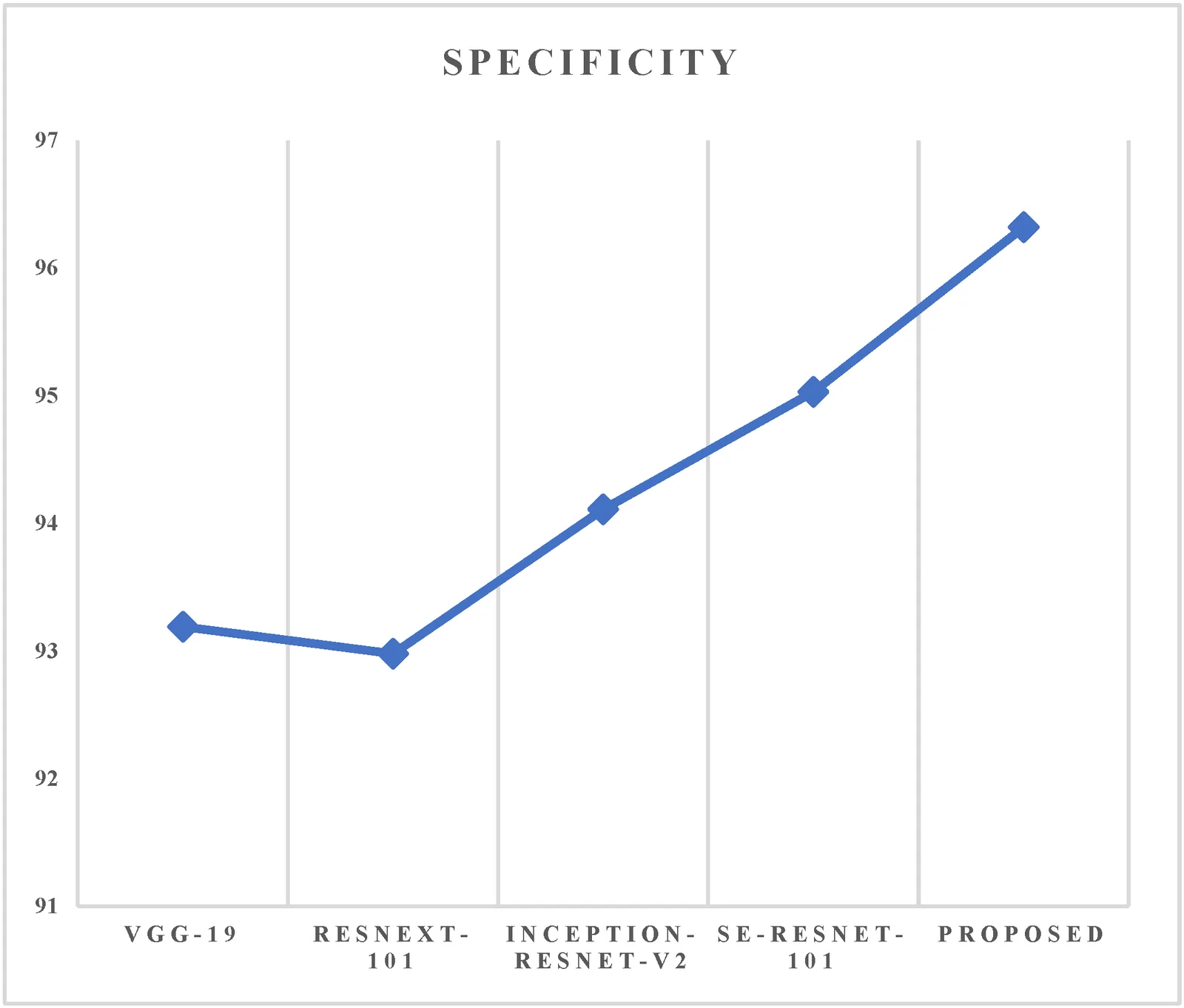
Comparison of image classification specificity.

**Figure 11:**
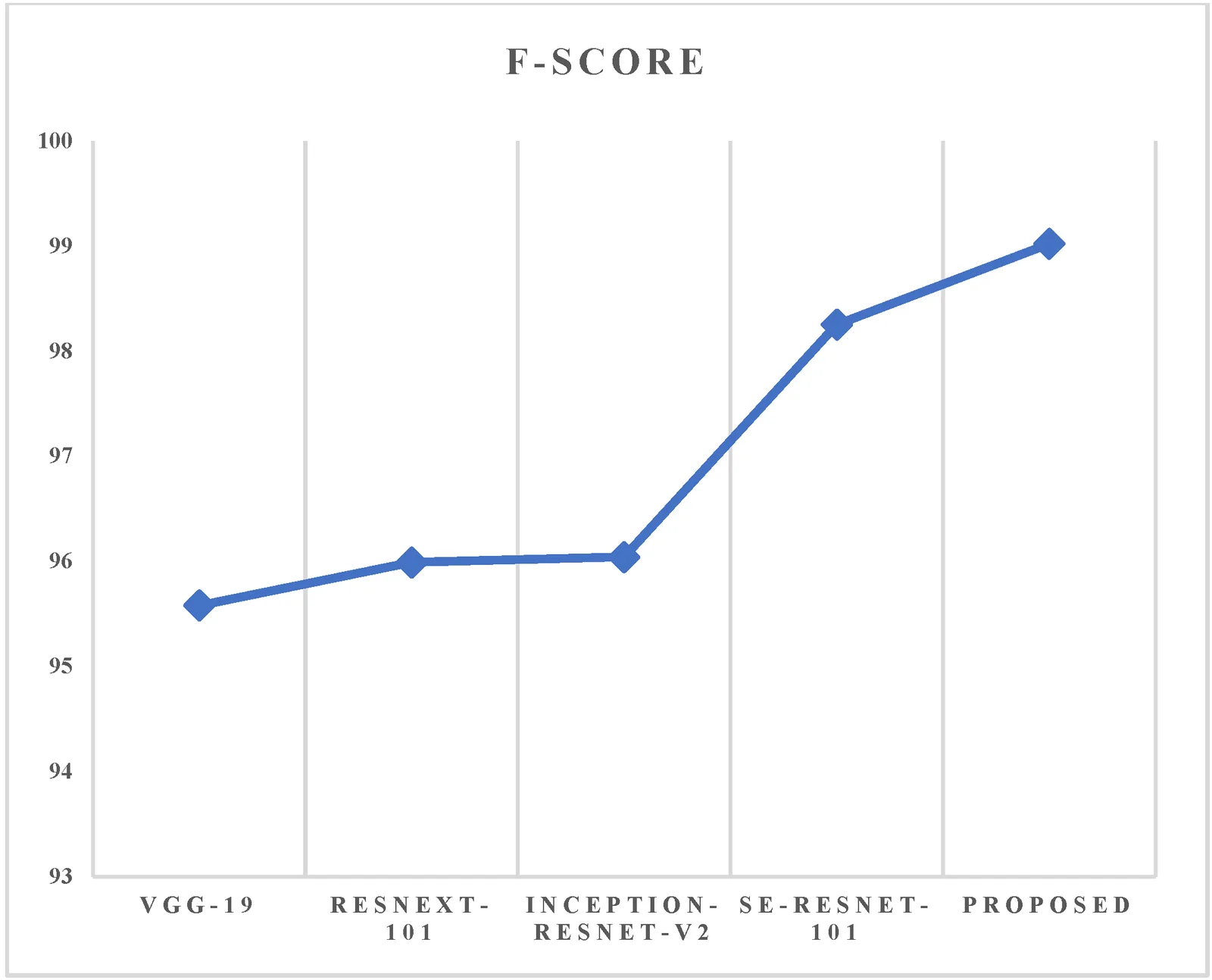
Comparison of image classification F-score.

The model has a specificity of 96.32 percent, which is 1.2 percent to 3.1 percent higher than the other models, as shown in Figure 10. The proposed model outperformed the other models in terms of F-score performance with a 99.02 percent F-score, which is 0.7 percent to 3.4 percent higher than that of the other models, as shown in Figure 11.

**Figure 12:**
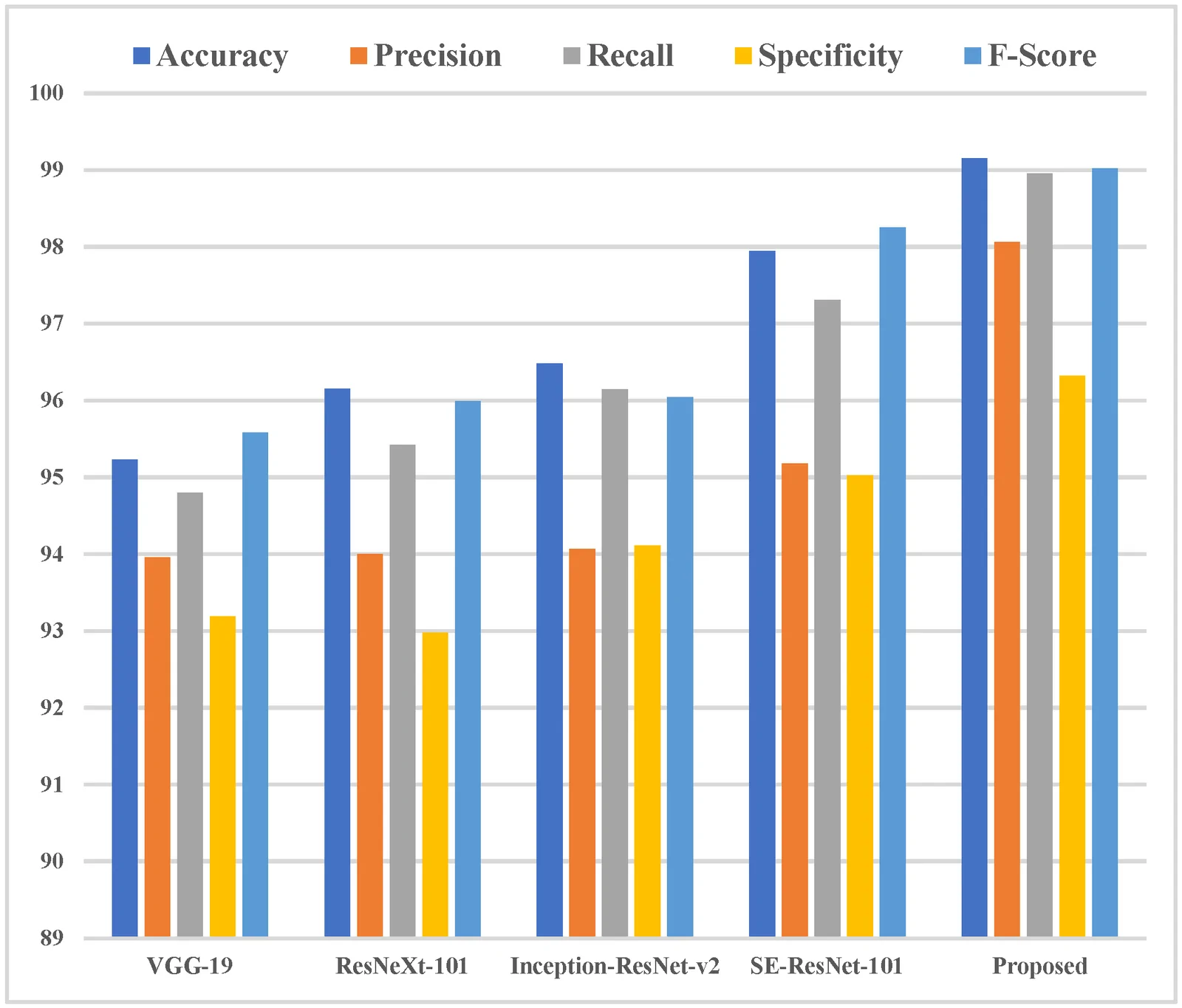
Comparison of entire performance analysis.

Figure 12 provides a graph of overall performance analysis. The performance of the SE-ResNet-101 and Inception-ResNet-v2 models was superior to that of other models, such as VGG-19 and ResNeXt-101. Table 4 demonstrates that the proposed hybrid Faster-RCNN with SE-ResNeXt-101 model is efficient for medical image classification. Both proposed models, the hybrid LDA-MALO and the hybrid Faster R-CNN with SE-ResNeXt-101, performed well in classifying heart disease data and were also appropriate for IoMT-based heart disease prediction.

## 5. Conclusions

Using machine learning algorithms, an IoMT-based heart disease prediction model was proposed in this research. The proposed model was tested in two stages. If the results of the first stage are accurate and efficient in predicting heart disease, stage two is unnecessary. In the first stage, medical data obtained from the patient’s body via sensors (wearables) were used for classification; in the second stage, echocardiogram images were used for classification. Both of these classification techniques were carried out, and the classification findings were verified for heart disease prediction. A hybrid linear discriminant analysis with modified ant lion optimization (HLDA-MALO) technique was used to classify sensor data. A hybrid Faster R-CNN with SE-ResNet-101 model was employed for echo image classification. To train the models, UCI repository datasets pertaining to heart disease, such as the Cleveland dataset and the echocardiogram dataset, were employed. The hybrid LDA-MALO technique detected normal sensor data with 96.85 percent accuracy and abnormal sensor data with 98.31 percent accuracy. The proposed hybrid Faster R-CNN with SE-ResNeXt-101 transfer learning model outperformed other models in identifying echocardiography images, with 98.06 percent precision, 98.95 percent recall, 96.32 percent specificity, a 99.02 percent F-score, and 99.15 percent maximum accuracy. In the future, Faster R-CNN may be enhanced with sophisticated deep transfer learning models such as SqueezeNet and EfficientNet, and larger datasets can be employed for improved analysis.

## Figures and Tables

**Table 1:** Descriptions of Cleveland dataset [7].

Name	Type	Description
**Age**	Continuous	Age
**Sex**	Discrete	1 = male; 0 = female
**Cp**	Discrete	Chest pain types: 1—typical angina, 2—atypical angina, 3—non-anginal pain, 4—asymptomatic
**Trestbps**	Continuous	Resting BP
**Chol**	Continuous	Serum cholesterols
**Fbs**	Discrete	Fasting blood sugar > 120 mg/dL: 1—true; 0—false
**Exang Continuous max. heart rates obtained**	Discrete	Exercises caused angina: 1—yes; 0—no
**Thalach**	Continuous	Max. heart pulse acquired
**Old peak ST**	Continuous	Depressions caused by exercises related to rest
**Slopes**	Discrete	The slopes of the peak exercise segment: 1—up sloping, 2—flat, 3—down sloping
**Ca**	Continuous	Total major vessel colored by fluoroscopy ranged from approximately 0 to 3
**Thal**	Discrete	3—normal, 6—fixed defects, 7—reversible defects
**Class**	Discrete	Diagnosis class: 0—no disease, 1—likely to have heart disease, 2—>1 3—>2 4—more likely have heart disease.
**Restecg**	Continuous	Resting electrocardiographic (ECG) results: value 0: normal; value 1: having ST-T wave abnormality (T wave inversions and/or ST elevation or depression of >0.05 mV); value 2: showing probable or definite left ventricular hypertrophy by Estes’ criteria.

**Table 2:** Descriptions of EchoNet-Dynamic Dataset.

Feature	Description
Videos	10,030
Image Height	112 pixels
Image Width	112 pixels
Resolution	112 × 112 pixels
View	Apical 4-Chamber (A4C)
Frames/Video	24–1002
Image Type	Grayscale
Videos	10,030
Image Height	112 pixels
Image Width	112 pixels
Resolution	112 × 112 pixels
View	Apical 4-Chamber (A4C)
Frames/Video	24–1002

**Table 3:** Comparison of normal and abnormal class subjects using hybrid LDA-MALO technique.

Data	Class	Accuracy	Precision	Recall	Specificity	F-Score
**Sensor (collected)**	Normal	96.85	95.10	97.04	94.46	95.23
**Cleveland dataset**		98.53	96.74	98.92	95.25	98.15
**Sensor**	Abnormal	98.31	96.48	98.83	97.52	97.98
**Cleveland dataset**		97.48	95.59	98.02	96.80	97.01

**Table 4:** Comparison of image classification performance.

Algorithm	Accuracy	Precision	Recall	Specificity	F-Score
**VGG-19**	95.23	93.96	94.80	93.19	95.58
**ResNeXt-101**	96.15	94.00	95.42	92.98	95.99
**Inception-ResNet-v2**	96.48	94.07	96.14	94.11	96.04
**SE-ResNet-101**	97.94	95.18	97.31	95.03	98.25
**Proposed model**	99.15	98.06	98.95	96.32	99.02

## Data Availability

The datasets used in this study are publicly available. The Heart Disease dataset used for the first-stage medical data classification is available from the UCI Machine Learning Repository at: https://archive.ics.uci.edu/dataset/45/heart+disease (accessed on 21 July 2021). The echocardiography dataset used for the second-stage image classification is the EchoNet-Dynamic dataset, which is publicly available at: https://echonet.github.io/dynamic/ (accessed on 21 July 2021).

## Code Availability

Custom code.
