# Correction: Manimurugan et al. Two-Stage Classification Model for the Prediction of Heart Disease Using IoMT and Artificial Intelligence. *Sensors* 2022, *22*, 476

**DOI:** 10.3390/s26195990

**Published:** 2026-09-22

**Authors:** S. Manimurugan, Saad Almutairi, Majed Mohammed Aborokbah, C. Narmatha, Subramaniam Ganesan, Naveen Chilamkurti, Riyadh A. Alzaheb, Hani Almoamari

**Affiliations:** 1 Industrial Innovation & Robotics Center, Faculty of Computers and Information Technology, University of Tabuk, Tabuk 47512, Saudi Arabia; s.almutairi@ut.edu.sa (S.A.); m.aborokbah@ut.edu.sa (M.M.A.); narmatha@ut.edu.sa (C.N.); 2 Department of Electrical and Computer Engineering, Oakland University, Rochester, MI 48309, USA; ganesan@oakland.edu; 3 Department of Computer Science and IT, La Trobe University, Melbourne 3086, Australia; n.chilamkurti@latrobe.edu.au; 4 Faculty of Applied Medical Sciences, University of Tabuk, Tabuk 47512, Saudi Arabia; ralzaheb@ut.edu.sa; 5 Faculty of Computer and Information Systems, Islamic University of Madinah, Medina 42351, Saudi Arabia; hani.almoamari@iu.edu.sa

## Text Correction

There were errors in the original publication [1]. A brief explanation was added to clarify the scientific record and to preempt any future misunderstandings regarding the methodology.

1. In Section 4.1 (Dataset Description) a correction has been made. The first stage of the medical data was classified based on the Cleveland Heart Disease dataset from the UCI Machine Learning Repository. The second stage image classification was done with the EchoNet-Dynamic dataset that was used to train and test the proposed Faster R-CNN with SE-ResNeXt-101 model. EchoNet-Dynamic is an open-source echocardiographic video database with annotated video and clinical data to support deep learning research. The old version of the manuscript used the UCI Echocardiogram dataset.

The correct Paragraph 2 in Section 4.1 appears below:

The description of the echocardiogram image dataset is presented in Table 2 with appropriate attributes. For the first-stage experiment, the selected features of the UCI Heart Disease dataset were used to develop and evaluate the HLDA-MALO classification model for distinguishing normal and abnormal cases. The medical sensor data collected through the proposed IoMT system was subsequently evaluated using the features considered during model development. The sensor data included measurements obtained from ECG, pulse oximeter, temperature, and blood-pressure sensors. For the second stage, the EchoNet-Dynamic dataset was used for echocardiography image classification. This dataset is publicly available and was used for the classification of echocardiographic images using the proposed hybrid Faster R-CNN with SE-ResNeXt-101 model. The echocardiography data were used to evaluate the capability of the proposed deep-learning model for distinguishing normal and abnormal cardiac images. Thus, the two stages of the proposed framework use two distinct publicly available datasets. The UCI Heart Disease dataset provides the medical attributes for the first-stage classification, whereas the EchoNet-Dynamic dataset provides the echocardiographic data for the second-stage image classification (Figure 4). The two datasets were analyzed independently according to their respective data modalities, and the resulting classification outcomes were used in the proposed two-stage heart disease prediction framework.

2. A correction has been made to Section 4.2, Paragraph 5. The correct paragraph appears below:

The proposed Faster R-CNN-SE-ResNeXt-101 model showed higher classification accuracy for classifying echocardiography images for heart disease prediction, as shown in Figure 7. The model achieved 99.15 percent accuracy, which is 1.2 percent to 3.9 percent higher than other approaches, such as VGG-19, ResNeXt-101, Inception-ResNet-v2, and SE-ResNet-101. The precision estimation was tabulated, indicating that the proposed model attained a higher precision value of 98.06 percent. The precision performance of the proposed model increased by 2.8 and 4.1 percent when compared to the other models, as shown in Figure 8. The reported results were computed using five-fold cross-validation, where the dataset was divided into five equal subsets. In each iteration, four folds were used for training, and one fold was used for testing, and the process was repeated five times. For example, fold accuracies such as 98.9%, 99.3%, 99.0%, 99.4%, and 99.15% were averaged to obtain the final accuracy of 99.15%. Similarly, precision, recall, specificity, and F-score values were also calculated for each fold and averaged to produce the final reported metrics.

## Table Correction

Table 2 in Section 4.1 has been updated accordingly. The correct Table 2 appears below:

**Table 2:** Descriptions of EchoNet-Dynamic Dataset.

Feature	Description
Videos	10,030
Image Height	112 pixels
Image Width	112 pixels
Resolution	112 × 112 pixels
View	Apical 4-Chamber (A4C)
Frames/Video	24–1002
Image Type	Grayscale
Videos	10,030
Image Height	112 pixels
Image Width	112 pixels
Resolution	112 × 112 pixels
View	Apical 4-Chamber (A4C)
Frames/Video	24–1002

## Back Matter Correction

The Data Availability Statement has been updated from “The authors confirm that the data supporting the findings of this research are available within the article” to “The datasets used in this study are publicly available. The Heart Disease dataset used for the first-stage medical data classification is available from the UCI Machine Learning Repository at: https://archive.ics.uci.edu/dataset/45/heart+disease (accessed on 21 July 2021). The echocardiography dataset used for the second-stage image classification is the EchoNet-Dynamic dataset, which is publicly available at: https://echonet.github.io/dynamic/ (accessed on 21 July 2021)”.

The authors state that the scientific conclusions are unaffected. This correction was approved by the Academic Editor. The original publication has also been updated.
